# In memory of Jean de Vellis (1935–2018)

**DOI:** 10.1186/s11689-019-9298-5

**Published:** 2019-12-16

**Authors:** Susan Y. Bookheimer, Harley I. Kornblum

**Affiliations:** Intellectual and Developmental Disabilities Research Center, Semel Institute for Neuroscience and Human Behavior and Department of Psychiatry and Biobehavioral Sciences, David Geffen School of Medicine at UCLA, 635 Charles E. Young Drive South, Los Angeles, CA 90095 USA

It is fitting to dedicate the Jean de Vellis memorial issue of *Journal of Neurodevelopmental Disorders* to research in glial biology: Jean was an early pioneer in this field, whose work has continued to shape the careers of outstanding investigators, many of whom have contributed to this issue. It may therefore seem surprising to those who did not know him personally to learn that Jean was actually trained as a plant biochemist. His love of agriculture and especially viticulture--the cultivation of grapevines--began in his early childhood. Jean (nobody who knew him called him Dr. de Vellis) was born to a French family in Tunisia and was raised on their citrus farm. Just before he would have attended elementary school, World War II broke out and his formal studies did not start until after the war, when he attended a Jesuit boarding school next to the ancient ruins of Carthage.

After receiving his baccalaureate, Jean attended the prestigious École Nationale Supérieure d’Horticulture located in Versailles. Jean then pursed his graduate studies at UCLA where he earned a PhD in plant biochemistry, studying the metabolism of bush bean roots. While earning his PhD, he met his future wife Phyllis, who introduced him to her neighbor, the future Director of the UCLA Brain Research Institute, Carmine Clemente, himself a pioneer in the neurosciences. Although Jean had no background in neuroscience, Carmine convinced Jean to join UCLA’s nascent neuroscience program, moving his studies of metabolism from beans to brains. The Atomic Energy Commission had established and funded a medical monitoring program for the Manhattan project scientists, directed by Stafford L. Warren, the founding Dean of the UCLA School of Medicine. At UCLA, Dr. Warren established the Laboratory of Nuclear Medicine and Radiation Biology at UCLA. Jean was recruited by Dr. Clemente as a faculty member in this Division, which subsequently become part of the Department of Energy Laboratory system.

Jean became a glial cell biologist through his early studies of the effects of radiation on brain development. It is now well known that white matter, in particular, is highly vulnerable to radiation, but this was not clear at the time. Jean began to study the composition of irradiated brains, and developed a collaboration with Harvey Herschman, a cell biology student at the University of California, San Diego, working on a presumed brain specific protein, S100. Jean examined the expression of this protein in cultured astrocytomas from rat (cell line C-6). These first experiments involved substantial development of new technologies such as creating immunodiffusion plates and placing proteins in wells with antibodies in gels. Jean sent Harvey samples of irradiated and normal rat brains during development, and Harvey assayed the proteins. Harvey ultimately moved to UCLA and the two became lifelong friends and collaborators, sharing adjacent space in Warren Hall for many years. Jean’s research gradually moved into the study of other extrinsic regulators of brain development and then, more specifically, glial development—a move that proved to be prophetic. As his work in the field developed, he became a leading expert on the culture of brain cell lines and the factors that promoted their growth. This work led to one of the most seminal studies and critical technical advances in the basic neurosciences: the culture of highly purified primary astrocytes and oligodendrocytes from rodent brain [1] which has been cited over four thousand times. This paper, along with a subsequent one with his graduate student, Rick Morrison [2] on the culture of primary astrocytes in chemically defined medium, became the standards in the field of mammalian brain cell culture. These reports opened the door to innumerable studies on the cellular properties and functions of these major cell types of the white matter, as well as on the cellular basis of developmental myelination. Many of the techniques and even media components for these procedures became commonly used in the culture of purified neurons neural stem cells and glioma cells. One of us (HIK), in fact, learned the methods directly from Dr. Morrison in the 1980’s and is still actively using them today. The de Vellis lab continued in its pursuit of developing and refining tissue culture methodology, with a methodological paper on the generation of oligodendrocytes from induced pluripotent stem cells published as recently as 2016 [3].

Although becoming a pioneer in now standard methodology in mammalian cell culture would be enough for many investigators, Jean’s real goals were to both understand normal brain development and to find treatments for developmental and acquired central nervous system disorders. Up until his death, Jean continued to pursue research on glial biology, focusing on the cellular basis for Canavan’s disease, a rare and devastating white matter disorder; he hoped to discover how cell transplantation might alleviate progression in this otherwise fatal neurodevelopmental condition. Jean’s record of publication is vast in its breadth and includes numerous gems in the most prestigious journals. They reflect a high degree of creativity and thought, as well as technical accomplishment.

Jean was a pillar of the neuroscience community at UCLA and on the national and international level. In 1970, UCLA was in the first group that was funded as an MRRC under the first director George Tarjan, a pioneer of mental retardation research. Jean was the named the Director in 1974 and held that post until 2015. Jean remained active as Associate Director until his retirement in 2018. Under Jean’s leadership, the MRRC Center grant was funded continuously from 1974 until the present. Over the course of Jean’s tenure as Director, he attracted and developed a high-level research environment bringing in leaders in basic developmental and translational neuroscience. Jean also used his position at Director to bring attention to developmental neuroscience to the leadership at UCLA.

On a National Level, Jean became a major voice in the promotion of research into developmental disorders, interacting extensively with leaders from other centers as well as with the leadership at the NIH. Amazingly, given his extensive responsibilities at UCLA, Jean was active in leadership roles in many organizations, including the International Society for Neurochemistry, The Institute for Developmental Neuroscience and Aging, the NICHD Mental Retardation Research Committee and the highly influential Christopher Reeve Paralysis Research Foundation. Jean was also active in organizing the Winter Brain Conference, where he was able to show off his considerable skiing skills. Jean served on multiple editorial boards, but is most noted for his work on the *Journal of Neuroscience Research*, where he was Editor-in-Chief for 16 years from 1999 to 2015. This journal became a strong publisher of many important articles in developmental neuroscience at a time when there were few options for many in the field.

While Jean’s intellect and scientific knowledge were enormous, the real key to much of his success was his warm personality, reflected in his mentorship style. Jean did not believe in distancing himself from his trainees. His students, postdocs, technicians and the many junior faculty for whom he was an unofficial mentor, automatically became part of his family. Jean made no distinction between his private life and his work life. Jean and Phyllis, his wife of 55 years, had three children, Phillipe and Genevieve, and daughter Gabrielle, who died tragically as a teenager. His mentees spent time with his family at their beautiful home in the Pacific Palisades, where he proudly displayed his stunning garden, reflecting his deep roots in agriculture and evoking memories of his childhood citrus farms.

While Jean officially retired 2 years before his death, he was an active emeritus professor. He remained engaged and cheerful, was frequently found in his office completing his last studies on Canavan Disease up until his passing on February 12, 2018. Jean’s family, friends and nearly one hundred colleagues attended his memorial service, and a memorial scientific symposium held in his honor in March of 2019. This memorial issue of the *Journal of Neurodevelopmental Disorders* vividly demonstrates the broad and lasting impact of Jean de Vellis’ life and career. He will be sorely missed.

## Data Availability

Not applicable
